# Self-growing environmentally responsive houses made from agricultural waste and fungal mycelia

**DOI:** 10.1093/synbio/ysac003

**Published:** 2022-02-08

**Authors:** Sonja Billerbeck

**Affiliations:** Molecular Microbiology, Groningen Biomolecular Sciences and Biotechnology Institute, University of Groningen, Groningen, The Netherlands

Mix the ingredients, pour them into a tin, and ‘bake’ at ambient temperature for 5 days. What sounds like instructions for a ready-made baking mix could soon become a way to grow your own home—or emergency shelter needed after a natural disaster (1).

While synthetic biology often focuses on using cells as factories to make molecules and nano-structures of interest, Rodrigo--Navarro *et al* focused on the cells themselves as the building blocks of macro-structure materials suitable for houses and shelters. This “engineered living material” (ELM) could be grown on demand, they are self-healing, responsive to environmental cues, and recyclable into new structures (2).

This macro-scale ELM was developed in a collaboration between the New York-based biomaterial company Ecovative Design and the laboratories of Prof. Harris Wang (Columbia) and Prof. Chris Voigt. The ‘recipe’ for the team’s ELM requires a mix of agricultural byproducts, water, flour and calcium sulfate, and the tree fungus *Ganoderma spec.* The fungus uses the agricultural waste for nutrition and structural support. Once mixed and cast into brick-shaped foldable paper moulds, the fungal mycelia glue the agricultural waste together into a dense material. In contrast to Ecovative’s standard process of ‘baking’ the ingredients at high temperature, which kills the fungus, McBee *et al* were able to desiccate the material at ambient temperature. In this state, the fungus rests but can be revived by moisturization. This allows casting of modular bricks that can later be grown together into larger 3D structures—like walls or shelters—without additional mortar. It also allows the material to self-heal if broken. The authors show that a broken brick could be regrown by placing the broken halves close to each other with the healed material retaining most of its original mechanical properties. Further, the material could be fully recycled by grinding it down and using it as inoculum to grow new bricks.

After developing this core living material, the team went one step further and equipped it with additional functions by adding an engineered bacterium that carries user-defined synthetic circuitry to the material mix. Instead of using an established, laboratory-tamed synthetic biology chassis such as *Escherichia coli*, which might have been outcompeted by the fungus, the authors performed a detailed microbiome analysis of the material, identifying and isolating a prevalent member, *Pantoea agglomerans*. They turned *P. agglomerans* into an engineerable chassis that could be reintroduced and maintained within the material. The authors then implemented a toy circuit distributed over two engineered strains of *P. agglomerans*. The first strain generated a volatile quorum sensing molecule (sender strain) that could be sensed and propagated through the material by a second strain (responder-propagator strain) that also created a fluorescent output that could be visualized under the microscope. As such, individual bricks could communicate with each other through their embedded engineered bacteria.

The authors developed a prototype material that might readily allow the growth of human-sized 3D-structures on demand from materials gathered on site. But important challenges remain, especially for the engineering components, such as containing the engineered components, strengthening the circuit output signals, and making structures robust to heavy rain or harsh sunlight. Will we soon be able to grow houses that change their outside hydrophobicity during heavy rain, which have built-in sun responsive UV filters, or that can detect harmful levels of pollutants present in the environment?

If the authors’ ambitions are realized, their easy-bake shelters could provide more warmth and satisfaction than the best cake.
